# Multidrug-resistant *Citrobacter freundii* ST139 co-producing NDM-1 and CMY-152 from China

**DOI:** 10.1038/s41598-018-28879-9

**Published:** 2018-07-13

**Authors:** Lang Yang, Peihan Li, Beibei Liang, Xiaofeng Hu, Jinhui Li, Jing Xie, Chaojie Yang, Rongzhang Hao, Ligui Wang, Leili Jia, Peng Li, Shaofu Qiu, Hongbin Song

**Affiliations:** 1 Academy of Military Medical Sciences, Beijing, China; 2 Institute for Disease Control and Prevention of PLA, Beijing, China

## Abstract

The emergence of carbapenemase-producing *Citrobacter freundii* poses a significant threat to public health worldwide. Here, we reported a *C. freundii* strain CWH001 which was resistant to all tested antimicrobials except tetracycline. Whole genome sequencing and analysis were performed. The strain, which belonged to a new sequence type ST139, showed close relationship with other foreign *C. freundii* strains through phylogenetic analysis. A novel variant of the intrinsic *bla*_CMY_ gene located on the chromosome was identified and designated as *bla*_CMY-152_. Coexistence of *bla*_NDM-1_ with *qnrS1* was found on a conjugative IncN plasmid, which had a backbone appearing in various plasmids. Other class A ESBL genes (*bla*_VEB-3_ and *bla*_TEM-1_) were also detected on two different novel plasmids. The emergence of multidrug-resistant *C. freundii* is of major concern, causing great challenges to the treatment of clinical infections. Great efforts need to be taken for the specific surveillance of this opportunistic pathogen.

## Introduction

*Citrobacter freundii*, a gram-negative bacterium of the *Enterobacteriaceae* family, is often the causative pathogen of a wide spectrum of nosocomial infections involving the respiratory tract^1^, urinary tract^2^ and bloodstream^3^. Previous studies have also reported its association with neonatal meningitis and brain abscess of high mortality^4^. Multidrug resistance in opportunistic pathogen *C. freundii* raised particular concern considering the severe dependence of immunocompromised patients on antibiotics^5^, and posed a significant threat to patient care and public health.

New Delhi metallo-β-lactamase 1 (NDM-1), a mediator of carbapenem resistance, had spread across different members of *Enterobacteriaceae*^6^ including *C. freundii* since its first identification in 2009^7^. The occurrence of *bla*_NDM-1_-positive *C. freundii* has been increasingly reported in China^8–11^, India^12,13^, Denmark^14^ and South Africa^15^. The majority of *C. freundii* with NDM-1 were often co-resistant to multiple antimicrobial agents, but usually remained susceptible to amikacin, gentamicin and fosfomycin^9–11^.

In this study, we report an NDM-1-producing *C. freundii* strain, which showed extensive resistance to nearly all tested antibiotics. Whole genome sequencing and analysis were performed to gain an insight into its genetic features and plasmid profiles.

## Results

### Microbiological and genetic features of strain CWH001

**Figure 1:**
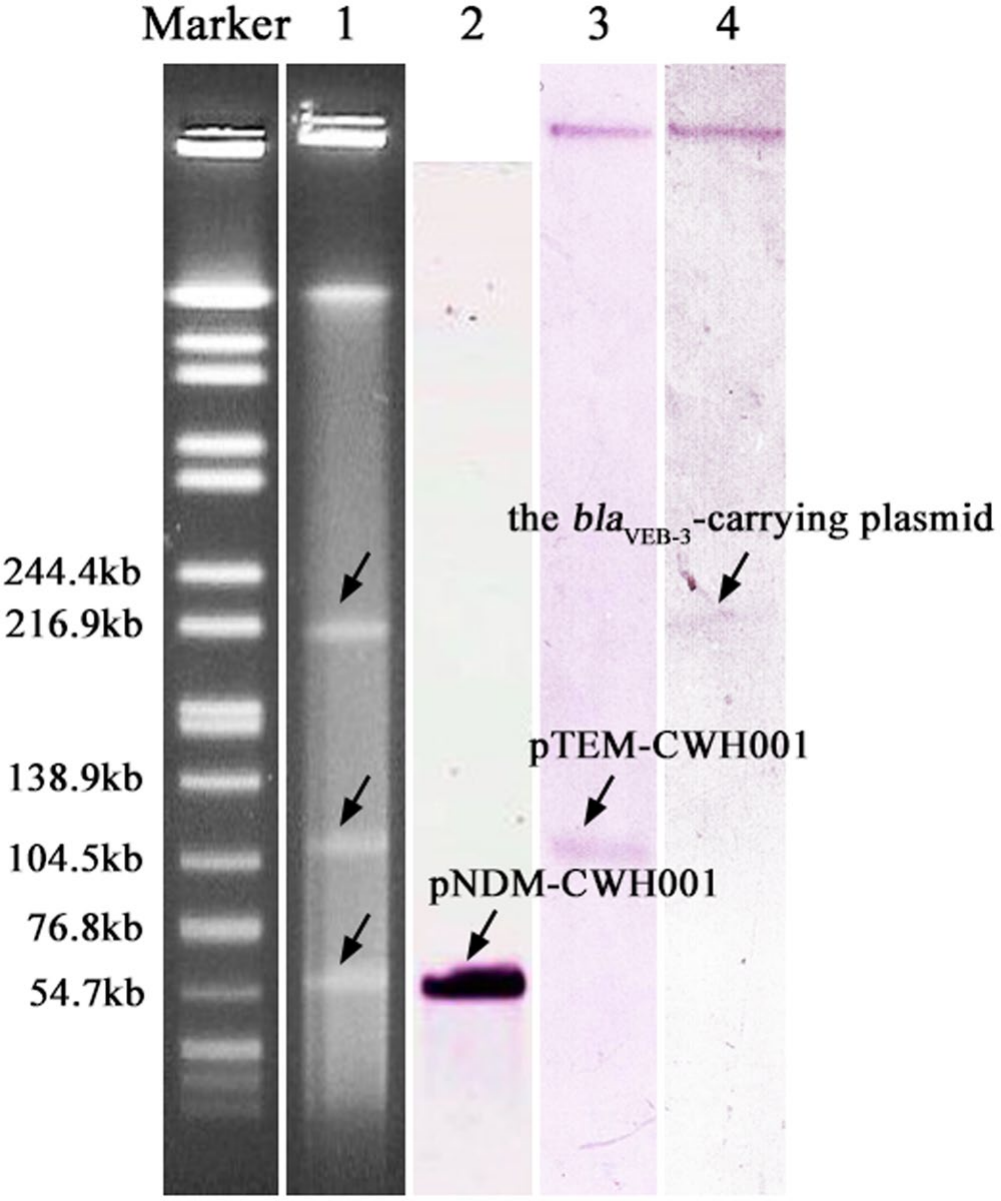
S1-PFGE pattern for strain CWH001 and southern blotting for the *bla*_NDM-1_, *bla*_TEM-1_ and *bla*_VEB-3_ genes. Lanes: Marker, *Salmonella* serotype Braenderup strain H9812 as a reference size standard; 1, PFGE result for S1-digested plasmid DNA of strain CWH001; 2–4, southern blot hybridization with the probes specific to *bla*_NDM-1_, *bla*_TEM-1_ and *bla*_VEB-3_, respectively. Full length S1-PFGE and southern blotting results are presented in Supplementary Fig. S1.

Strain CWH001 was recovered from the blood sample of a patient through routine surveillance in Wuhan, China, in 2014. The strain was identified as *C. freundii* using Vitek 2 compact system and confirmed by 16S rDNA sequencing. CWH001 was resistant to nearly all tested antibiotics including aminoglycosides, cephalosporins, carbapenems, fluoroquinolones and sulfonamides, but remained susceptible to tetracycline (Table 1). PCR amplification and sequencing confirmed the presence of *bla*_NDM-1_. S1 pulsed field gel electrophoresis (PFGE) showed that CWH001 contained three different plasmids (~60 kb, ~105 kb and ~220 kb) (Fig. 1). Southern blotting revealed that the *bla*_NDM-1_ gene was carried by the ~60 kb plasmid, which was transferable to *Escherichia coli* J53 at a high transfer frequency of 2.21 × 10^−2^ per donor cell. The transconjugants acquired resistance to amoxicillin-clavulanic acid, piperacillin, imipenem and meropenem. Interestingly, subsequent sequencing and southern blotting revealed that there existed the *bla*_VEB-3_ gene on the ~220 kb plasmid, which was transferred to the transconjugants simultaneously. A BLAST search indicated that the 3.2 kb *bla*_VEB-3_-carrying contig was composed of a novel combination of *Klebsiella pneumoniae* JM45 plasmid p1 (CP006657, unpublished) and uncultured bacterium plasmid pKAZ5^16^. The presence of the *bla*_NDM-1_ and *bla*_VEB-3_ genes in the transconjugants was further confirmed by PCR amplification and sequencing.

**Table 1:** Antibiotic susceptibilities of *C. freundii* strain CWH001 and the *E. coli* J53 transconjugants.

Antimicrobial	MIC (μg/ml)	
	CWH001	J53 (the transconjucant)
Amoxicillin-clavulanic acid	≥32	≥32
Piperacillin	≥128	≥128
Cefazolin	≥64	≥64
Ceftazidime	≥64	≥64
Ceftriaxone	≥64	≥64
Cefepime	≥64	16
Aztreonam	16	≤1
Imipenem	≥16	≥16
Meropenem	8	8
Amikacin	≥64	4
Gentamicin	≥16	≤1
Ciprofloxacin	≥4	1
Levofloxacin	≥8	1
Tetracycline	4	2
Nitrofurantoin	128	≤16
Sulfamethoxazole-trimethoprim	≥320	≤20

In addition to *bla*_NDM-1_ and *bla*_VEB-3_, other resistance genes were also identified in strain CWH001 including *bla*_TEM-1_, *qnrS1*, *dfrA12*, *armA*, *fosA3*, *mphA*, *sul1*, *aac(3)-IId* and a novel variant of the *bla*_CMY_ gene. Analysis of the deduced protein sequence of the *bla*_CMY_ variant revealed a single amino acid substitution at position 22 (Thr → Ala) relative to that of CMY-41. This variant protein was designated CMY-152 (http://www.lahey.org/Studies/webt.asp). BLAST search and southern blotting revealed the *bla*_CMY-152_ gene, together with its regulator gene *ampR* flanked by the upstream *frd* genes and the downstream *blc* gene, was located on the chromosome.

### Molecular typing and phylogenetic analysis

**Figure 2:**
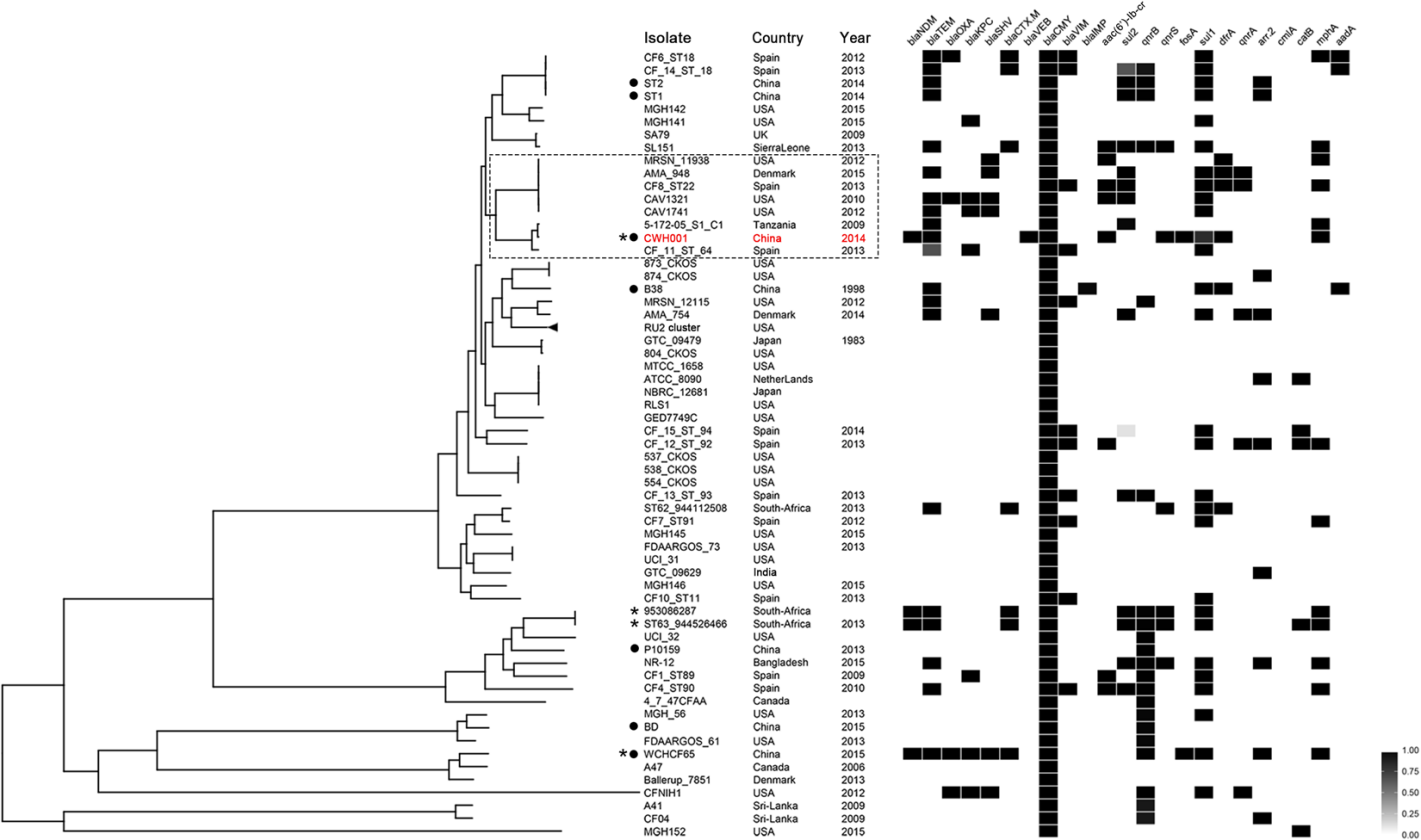
Phylogenetic tree and resistance gene profile of *C. freundii* strain CWH001 with other 84 available *C. freundii* genomes from GenBank. Strain CWH001 is indicated in red. Isolates from China are marked with a solid circle. NDM-harboring isolates are marked with an asterisk. The CWH001-including cluster is indicated with the dashed lines. Twenty-five closely related clones from the same geographical source (Houston, USA) are indicated as the RU2 cluster. Distribution of resistance genes is indicated by the heatmap according to the legend, which reflects percentage coverage of each gene sequence.

The NDM-1-producing *C. freundii* CWH001 did not belong to an existing sequence type and was assigned to a new ST, ST139, using the multi-locus sequence typing (MLST) web server. Phylogenetic analysis revealed a high degree of genetic diversity of 84 available *C. freundii* genomes with that of CWH001. CWH001 was clustered into clades with overseas strains, and had a close relationship with strain 5-172-05_S1_C1 from Tanzania (Fig. 2). Only 543 SNPs were detected between the chromosomes of strain CWH001 and 5-172-05_S1_C1. However, CWH001 fell into different clades and showing distant phylogenetic relationship to other domestic strains. Sequence alignments revealed the average nucleotide identity (ANI) between CWH001 and other isolates from China ranged from 92.20% to 98.52%, while the ANI between CWH001 and 5-172-05_S1_C1 was 99.50%, indicating a different evolutionary pathway of CWH001 from other domestic strains in China.

### Characterization of *bla*_NDM-1_-carrying plasmid pNDM-CWH001

**Figure 3:**
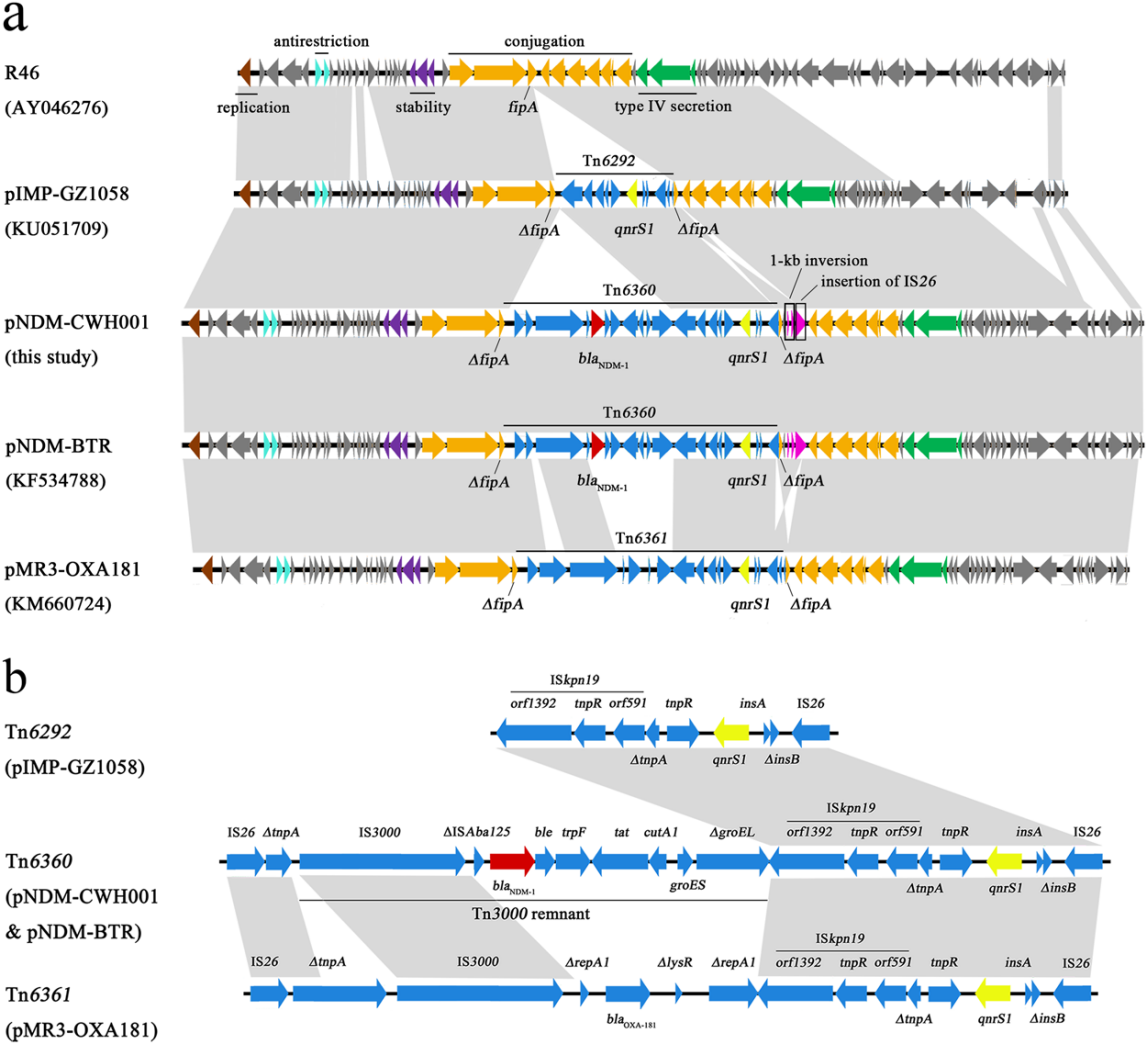
The comparative schematic diagram of (**a**) plasmids R46, pIMP-GZ1058, pNDM-CWH001, pNDM-BTR and pMR3-OXA181; (**b**) the accessory modules Tn*6292* in pIMP-GZ1058, Tn*6360* in pNDM-CWH001 and pNDM-BTR, and Tn*6361* in pMR3-OXA181. The open reading frames are indicated by arrows. The brown, cyan, purple, orange and green arrows represent genes associated with replication, antirestriction, stability, conjugation and type IV secretion system, respectively. The *bla*_NDM-1_ gene is shown in red. The *qnrS1* gene is shown in golden. The accessory modules are shown in blue. The 1-kb inversion region and insertion of IS*26* are shown in pink. Other genes of the backbone are shown in dark gray. Homology regions among different plasmids are denoted by light gray.

The 59-kb plasmid carrying *bla*_NDM-1_ was completely assembled and designated as pNDM-CWH001. pNDM-CWH001 belonged to the incompatibility type IncN. BLAST search against NCBI revealed that pNDM-CWH001 showed 100% coverage and >99% identity to the *E. coli* plasmid pNDM-BTR^17^ from China. Two single nucleotide deletions located within *virB4* and *virB8*, respectively, were identified in pNDM-BTR. pNDM-CWH001 consisted of a *bla*_NDM-1_-containing transposon Tn*6360* and a 42.3-kb backbone (Fig. 3a). Tn*6360* was composed of an accessory region carrying *bla*_NDM-1_ and an intact Tn*6292* element carrying *qnrS1* (Fig. 3b). The accessory region comprised an IS*26*, a 427-bp truncated *tnpA*, and an 8.3-kb Tn*3000* remnant (IS*3000*-ΔIS*Aba125*-*bla*_NDM-1_-*ble*-*trpF*-*tat*-Δ*cutA1*-*groES*-Δ*groEL*). Compared with the prototype Tn*3000*^18^, the remnant had undergone a deletion of the second copy of IS*3000* together with the truncation of *groEL* in the 3′ extremity, suggesting a possible transposition event. The transposon Tn*6292* had a quinolone resistance genetic platform organized as IS*26*-*qnrS1*-IS*kpn19*, which has been repeatedly reported in previous plasmids^19,20^ and was likely introduced due to the inter-plasmid transfer as a transposable element^21^.

The backbone of pNDM-CWH001 also presented >98% identity to those of pMR3-OXA181^22^ (100% coverage) and pIMP-GZ1058^23^ (92% coverage). The backbone contained a set of core genes for plasmid replication (*repA*), conjugation (*tra* genes), stability (*stdB*), antirestriction (*ardA* and *klcA*) and type IV secretion system (*virB* genes). However, there existed an inversion of a 1-kb region in plasmid pNDM-CWH001 and pNDM-BTR, which encoded aldehyde dehydrogenase and transcriptional regulator. An additional IS*26* was inserted following this inversion region. The *bla*_NDM-1_-carrying transposon Tn*6360* was integrated into the *fipA* gene, which was interrupted into two fragments in pNDM-CWH001 compared to plasmid R46^24^ and may serve as a “hotspot” for insertion of transposable elements.

### Genetic features of plasmid pTEM-CWH001

The *bla*_TEM-1_ gene was located on a novel plasmid designated as pTEM-CWH001, which had the length of 107,391 bp and comprised a combination of *C. freundii* plasmid p112298-KPC^9^ and *Salmonella enterica* plasmid pF8475^25^. pTEM-CWH001 could not be assigned to any known incompatibility group. The deduced replication initiator RepA presented >98% amino acid similarity with various IncFII family RepA proteins from *Citrobacter*. An insertion of IS*Ec42* between conjugal transfer genes *tra* and *trb* were observed, which was likely to impair the expression of the *trbABC* operon and may result in a non-transferable plasmid. pTEM-CWH001 harbored a Tn*21*-like structure bound by the transposition genes (*tnpAR*) and the *mer* operon in the 5′ and 3′ portion, respectively. The *bla*_TEM-1_ gene and an insertion sequence IS*Cfr1* were located upstream of the Tn*21*-like structure. Compared with the prototype Tn*21*, this structure had undergone the replacement of *aadA1* by *dfrA1*2 and an insertion of a macrolide resistance operon organized as *mphA*-*mrx*-*mphR* in the class 1 integron In*2*, suggesting possible frequent transposition events.

## Discussion

The ability to produce NDM-1 carbapenemases has been acquired by diverse *Enterobacteriaceae* species and posed a significant threat to public health. Our study identified a *bla*_NDM-1_-positive *C. freundii* isolate with coexistence of other multiple resistant determinants (*bla*_VEB-3_, *bla*_TEM-1_ and *bla*_CMY-152_) and provided detailed genetic characteristics of the NDM-1-carrying IncN plasmid pNDM-CWH001. Plasmids belonging to the IncN group are typically broad-host-range and self-conjugative^26^. The high transfer frequency of pNDM-CWH001 demonstrated its great potential to transfer across species. The resistance-determining region in those pNDM-CWH001-like plasmids was all inserted within the *fipA* gene. Interestingly, the *fip*A-encoded protein was reported to inhibit the conjugal transfer of some plasmids^27^. The interruption of the *fipA* gene could facilitate the ability of the plasmids of the plasmids to accumulate in diverse hosts and may serve as a “hotspot” for integration of mobile elements. Comparative analysis revealed that the acquisition of Tn*6292* and the Tn*3000* remnant might be subsequently integrated into pNDM-CWH001-like plasmids, highlighting the urgency of further surveillance and genetic analysis of such flexible mobile units for better understanding of extensive resistance dissemination.

Recent studies have reported the simultaneous presence of multiple resistance genes in *C. freundii* strains isolated in China^8,9,11^. However, CWH001 showed long-distance dispersals from other *C. freundii* isolates in China and gained some resistance determinants (*bla*_VEB-3_ and *fosA3*) that were rarely identified in other *C. freundii* isolates. Previous study has reported *C. freundii* strain WCHCF65 from China clustered with strains from Denmark^8^. Phylogenetic analysis revealed that domestic *C. freundii* isolates showed close relationship with overseas ones but fell into distinct clusters, indicating different evolution and dissemination route. Plasmid pNDM-BTR was isolated from Beijing in 2013. Though lacking of epidemiological association, the close spatial and temporal proximity between pNDM-CWH001 and pNDM-BTR in China suggested possible dissemination of this novel plasmid, and more attention should be devoted to monitoring the epidemic spread of such *bla*_NDM-1_-carrying IncN plasmids among *Enterobacteriaceae*.

In summary, our study characterized a multidrug-resistant *C. freundii* isolate harboring multiple ESBL-encoding genes. Strain CWH001 belonged to a novel sequence type ST139 with a self-transferable plasmid pNDM-CWH001, which may facilitate the *bla*_NDM-1_ gene dissemination. Phylogenetic analysis revealed that CWH001 had different origin from domestic isolates but gained multidrug resistance. Our findings further emphasize the threat of NDM-1 carbapenemase circulation among diverse species, and urgent actions should be taken to control the potential rapid spread of such plasmids.

## Materials and Methods

### Bacterial isolation and identification

The *bla*_NDM-1_-positive *C. freundii* strain CWH001 was recovered from the blood sample of a 63-year-old male patient through routine surveillance in Wuhan, China, in 2014. The species level identification was performed by using Vitek 2 compact system (bioMérieux, France) and confirmed by 16S rDNA sequencing^28^. The presence of genes encoding carbapenemases and ESBLs was determined by PCR and sequencing^29–31^. The entire *bla*_NDM_, *bla*_TEM_, *bla*_VEB_ and *bla*_CMY_ genes were amplified with previously described primers^32–35^. Positive PCR results were further confirmed by sequencing. The informed consent was obtained from the patient. All experimental protocols were approved by Institutes of Military Medicine, Academy of Military Sciences. The methods were carried out in accordance with relevant guidelines.

### Antimicrobial susceptibility testing

The minimal inhibitory concentrations (MICs) of amoxicillin/clavulanic acid (AMC), piperacillin (PIP), cefazolin (FAZ), ceftazidime (CAZ), ceftriaxone (CTR), cefepime (FEP), aztreonam (AZT), imipenem (IMI), meropenem (MEC), amikacin (AMI), gentamicin (GEN), ciprofloxacin (CIP), levofloxacin (LVX), tetracycline (TET), nitrofurantoin (NIT) and sulfamethoxazole/trimethoprim (SXT) were determined by Vitek 2 compact system (BioMérieux, France) following the manufacturer’s instructions. The results were interpreted following the Clinical and Laboratory Standards Institute (CLSI) guidelines^36^.

### Southern blotting and Conjugation experiment

Genomic DNA from strain CWH001 was prepared in agarose plugs and digested with the S1 endonuclease (Takara, Dalian, China). DNA fragments were separated by PFGE through a CHEF-DR III system (Bio-Rad, Hercules, USA). The plasmid DNA was transferred to a positively charged nylon membrane (Roche) and hybridized with the digoxigenin-labeled probes specific to *bla*_NDM-1_, *bla*_TEM-1_, *bla*_VEB-3_ and *bla*_CMY-152_.

Conjugation experiment was carried out by broth and filter mating using the clinical strain CWH001 as donors and azide-resistant *E. coli* strain J53 as the recipient. The donor and recipient cultures were mixed at a ratio of 1:3 in LB broth and incubated at 37 °C for 18 hours. The mixture was inoculated into MacConkey agar plates containing 4 μg/ml meropenem and 150 μg/ml sodium azide. The transconjugants were selected after 12 h of incubation. Horizontal transferability of drug resistance was assessed by antimicrobial susceptibility testing and the transconjugants carrying resistant markers (*bla*_NDM-1_, *bla*_VEB-3_) were confirmed by PCR amplification.

### Whole genome sequencing and phylogenetic analysis

Total DNA was extracted from cultured bacterium using the QIAamp DNA minikit (Qiagen, Inc., Valencia, CA). Sequencing was carried out using an Illumina HiSeq. 2500 platform with a 350-bp insert size at Novogene Company (Beijing, China). The genome was assembled *de novo* using SOAPdenovo (v2.04)^37^ with an average 110-fold coverage. Scaffolding and gap filling were performed using SSPACE and GapFiller^38,39^. Plasmids pNDM-BTR, p112298-KPC and pF8475 were selected as reference. Gaps were closed using reference-guided assembly and manually checked by re-mapping raw reads against the plasmids. Genome sequences were annotated using RAST^40^. Plasmid replicon type was identified using PlasmidFinder^41^ (*Enterobacteriaceae*).

Seven housekeeping genes (*arca*-*aspc*-*clpx*-*dnag*-*fadd*-*lysp*-*mdh*) were extracted from the genome of CWH001 and used in MLST typing through the MLST web server^42^. Genome sequences of 84 currently available *C. freundii* isolates were downloaded from the NCBI database for phylogenetic analysis (accessed 12th June 2017). *C. freundii* strain B38 (GenBank accession number CP016762) was used as the reference for comparison. Reads mapping was performed using BWA (v0.7.12)^43^. SNPs were identified using SAMtools (v1.3)^44^. The resulting 27366 SNPs were concatenated and aligned to construct the Maximum-Likelihood phylogenetic tree using RAxML (v8.2.4) with the general time reversible (GTR) model and a gamma distribution^45^. ANIs between CWH001 and other genomes were calculated using JSpeciesWS^46^ to evaluate the genome similarity.

### Nucleotide sequence accession number

The shotgun whole genome sequence of strain CWH001 and complete sequence of plasmids pNDM-CWH001 and pTEM-CWH001 have been deposited in NCBI GenBank under accession number PEHH00000000.

## Electronic supplementary material

Supplementary Information
