# Supplementary material for: Multidrug-resistant Citrobacter freundii ST139 co-producing NDM-1 and CMY-152 from China
Source: Sci Rep. 2018 Jul 13;8:10653. doi: 10.1038/s41598-018-28879-9 (PMC6045649; doi:10.1038/s41598-018-28879-9)
Supplement: Supplementary file 1 — Supplementary Information [file 41598_2018_28879_MOESM1_ESM.pdf]

Supplementary information

Multidrug-resistant *Citrobacter freundii* ST139 co-producing NDM-1 and CMY-152 from China

Lang Yang[1,2#], Peihan Li[1,2#], Beibei Liang[1,2#], Xiaofeng Hu[2#], Jinhui Li[2], Jing Xie[2], Chaojie Yang[2], Rongzhang Hao[2], Ligui Wang[2], Leili Jia[2], Peng Li[2*], Shaofu Qiu[2*] AND Hongbin Song[2*]

[1]Institutes of Military Medicine, Academy of Military Sciences, Beijing, China

[2]Institute for Disease Control and Prevention of PLA, Beijing, China

[#]These authors contributed equally to this work.

[*]Address correspondence to Peng Li, jiekenlee@126.com, Shaofu Qiu, qiushf0613@hotmail.com, or Hongbin Song, hongbinsong@263.net

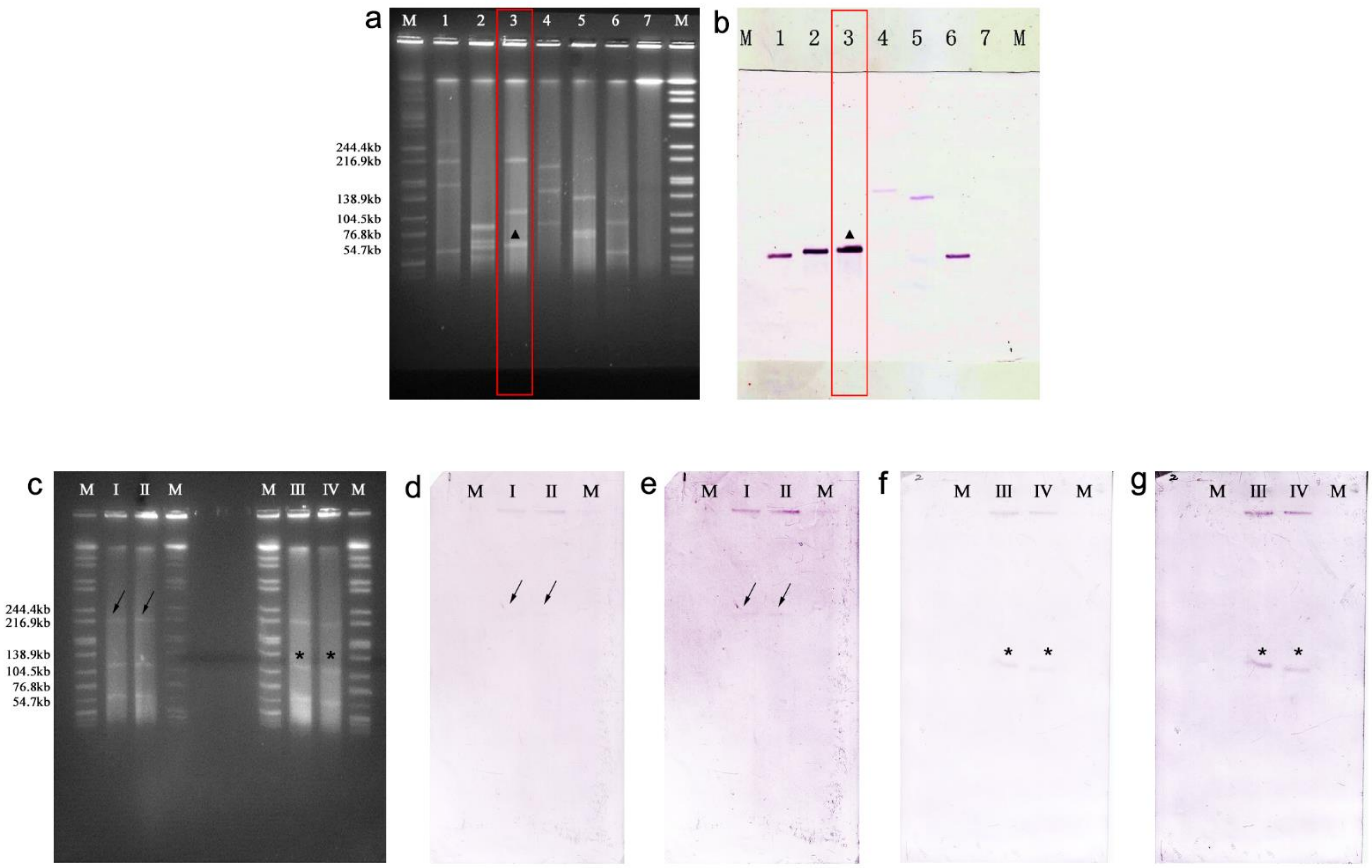

Supplementary Figure S1. S1-PFGE pattern for strain CWH001 and southern blot hybridization with the probe specific to *bla*$_{\text{NDM-1}}$, *bla*$_{\text{TEM-1}}$ or *bla*$_{\text{VEB-3}}$. The triangle, asterisk and arrow indicate the *bla*$_{\text{NDM-1}}$-, *bla*$_{\text{TEM-1}}$- and *bla*$_{\text{VEB-3}}$-positive signals, respectively. (a) Lane M: marker, *Salmonella* serotype Braenderup strain H9812 as a reference size standard; Lane 3: S1-PFGE pattern for strain CWH001; other lanes indicate the S1-PFGE patterns for other strains not related to this study. (b) Lane 3 in red rectangle presents the southern blot hybridization with the probe specific to *bla*$_{\text{NDM-1}}$ for strain CWH001. (c) Lane M: marker, *Salmonella* serotype Braenderup strain H9812 as a reference size standard; Lane I, II, III and IV: S1-PFGE pattern for strain CWH001. (d) The southern blot hybridization with the probe

specific to *bla*$_{VEB-3}$ for strain CWH001. (e) The high-contrast image of (d). (f) The southern blot hybridization with the probe specific to *bla*$_{TEM-1}$ for strain CWH001. (g) The high-contrast image of (f).
